# Molecular and Cytogenetic Characterization of New Wheat—*Dasypyrum breviaristatum* Derivatives with Post-Harvest Re-Growth Habit

**DOI:** 10.3390/genes6041242

**Published:** 2015-11-27

**Authors:** Hongjun Zhang, Guangrong Li, Donghai Li, Dan Gao, Jie Zhang, Ennian Yang, Zujun Yang

**Affiliations:** 1 School of Life Science and Technology, University of Electronic Science and Technology of China, Chengdu 610054, China; E-Mails: hongjunzh01@sina.com (H.Z.); ligr28@uestc.edu.cn (G.L.); lidonghaixy@163.com (D.L.); gaodangaodan2010@163.com (D.G); christine219@163.com (J.Z.); 2 Crop Research Institute, Sichuan Academy of Agricultural Sciences, Chengdu 610066, China; E-Mail: yangennian@126.com

**Keywords:** *Dasypyrum breviaristatum*, fluorescence *in situ* hybridization, molecular markers, wheat

## Abstract

A novel *Dasypyrum* species, *Dasypyrum breviaristatum*, serves as a valuable source of useful genes for wheat improvement. The development and characterization of new wheat—*D. breviaristatum* introgression lines is important to determine the novel gene(s) on specific chromosome(s). We first used multi-color fluorescence *in situ* hybridization (FISH) to identify the individual *D. breviaristatum* V^b^ chromosomes in a common wheat—*D. breviaristatum* partial amphiploid, TDH-2. The FISH patterns of *D. breviaristatum* chromosomes were different from those of *D. villosum* chromosomes. Lines D2146 and D2150 were selected from a cross between wheat line MY11 and wheat—*D. breviaristatum* partial amphiploid TDH-2, and they were characterized by FISH and PCR-based molecular markers. We found that D2150 was a monosomic addition line for chromosome 5V^b^ of *D. breviaristatum*, while D2146 had the 5V^b^L chromosome arm translocated with wheat chromosome 5AS. Molecular marker analysis confirmed that the introduced *D. breviaristatum* chromosome 5V^b^L translocation possessed a duplicated region homoeologous to 5AS, revealing that the 5AS.5V^b^L translocation may not functionally compensate well. The dwarfing and the pre-harvest re-growth habits observed in the wheat—*D. breviaristatum* chromosome 5V^b^ derivatives may be useful for future development of perennial growth wheat lines.

## 1. Introduction

The genus *Dasypyrum* (or *Haynaldia*) consists of two species, *Dasypyrum villosum* and *D. breviaristatum.* Cytological and molecular evidence suggest significant genomic diversification between the two species, and therefore the genome symbols of *D. villosum* and *D. breviaristatum* were assigned to V and V^b^, respectively [1,2]. Recently, Baum *et al.* [3] suggested the genome constitution of tetraploid *D. breviaristatum* as VVV^b^V^b^ (2*n* = 4*x* = 28) based on the evolutionary analysis of the nr5S DNA multi-gene family. Both *Dasypyrum* species displayed several agronomical important traits including those of disease resistance, high protein quality and drought tolerance, which offer valuable resources for wheat improvement [4,5]. The *D. villosum* species has been extensively hybridized to wheat, and several disease resistance genes have been successfully transferred to wheat [6,7,8]. With the aim to transfer useful genes from *D. breviaristatum* into wheat, we produced a wheat—*D. breviaristatum* partial amphiploid and several wheat—*D. breviaristatum* introgression lines by chromosome manipulation [9,10,11].

Precise identification of the alien chromosomes and wheat-alien recombinant chromosomes is essential for investigation of evolution and utilization of novel chromatin in wheat breeding. Chromosome C-banding and fluorescence *in situ* hybridization (FISH) are powerful techniques to visualize alien chromatin in wheat-alien hybrids [12,13]. The large heterochromatic C-bands of *D. villosum* chromosomes enable the identification of the *D. villosum* chromosomes and their rearrangement in the wheat background [14,15]. Recently, Zhang *et al.* [16] established a FISH karyotype of *D. villosum* chromosomes by probes of pSc119.2, pAs1, 45S and 5SrDNA. Meanwhile, the simple sequence repeat (GAA)*_n_* can be used as a FISH probe to characterize the individual *D. villosum* chromosomes [17]. However, *D. breviaristatum* chromosomes displayed less telomeric heterochromatin and generally had different C-banding patterns compared to those of *D. villosum* [18] The detailed karyotype of *D. breviaristatum* chromosomes needs to be established by molecular and cytogenetic methods.

Development of “perennial wheat” has been proposed as a potential method for sustainability of agricultural production, food security, and environmental quality [19]. Many Triticeae species have been used as donors of perennial growth habit to improve wheat [20,21]. As a perennial *Dasypyrum* species, *D. breviaristatum* has a strong perennial character with a post-harvest regrowth (PHR) habit, which could be transferred to a wheat background. Here we aimed to establish the karyotype of *D. breviaristatum* chromosomes in a wheat background, and characterize the novel wheat—*D. breviaristatum* introgression lines by using multicolor-fluorescence *in situ* hybridization and molecular markers.

## 2. Materials and Methods

### 2.1. Plant Materials

*D. breviaristatum* accession PI 546317 (genome VVV^b^V^b^, 2*n* = 4*x* = 28) was obtained from the National Small Grains Collection at Aberdeen, Idaho, USA. The wheat—*D. breviaristatum* partial amphiploid TDH-2 (genome AABBV^b^V^b^, 2*n* = 6*x* = 42) was as described by Yang *et al.* [9]. *Triticum turgidum* cv. Jorc-69- *D. villosum* amphiploid ABV (genome AABBVV, 2*n* = 6*x* = 42) was developed and provided by Prof. Hua-Ren Jiang at Sichuan Agricultural University, China [22]. Line D2146 and D2150 was obtained from the BC_1_F_4_ generation of a cross between wheat line MY11 and TDH-2.

### 2.2. Fluorescence in Situ Hybridization (FISH)

Seedling root tips were collected and pretreated in water at 0 °C for 24 h and fixed in ethanol-acetic acid (3:1) for conventional squashes. The nitrous oxide treated root-tip followed by enzyme digested drop method was also reported by Tang *et al.* [23]. FISH with the LTR probe pDbH12 was used to detect the *Dasypyrum* genome in a wheat background as reported by Yang *et al.* [24]. The synthesized probes Oligo-pSc119.2, Oligo-pTa535, Oligo-(GAA)_6_ were used in the FISH analysis [23]. The hybridization and detection protocols were as described by Fu *et al.* [25]. Microphotographs of FISH chromosomes were taken with an Olympus BX-51 microscope equipped with a DP-70 CCD camera.

### 2.3. Molecular Marker Analysis

DNA was extracted from young leaves of *D. breviaristatum*, TDH-2, ABV, lines D2146, D2150 and *Triticum aestivum* cv. “Chinese Spring” (CS). PCR-based Landmark Unique Gene (PLUG) primers and EST based primers were designed according to Ishikawa *et al.* [26] and Fang *et al.* [27], respectively. Polymerase chain reaction (PCR) was performed in an Icycler thermalcycler (Bio-RAD Laboratories, Emeryville, CA, USA) in a 25 μL reaction, containing 10 mmol Tris-HCl (pH 8.3), 2.5 mmol MgCl_2_, 200 μmol of each dNTP, 100 ng template DNA, 0.2 U Taq polymerase (Takara, Japan) and 400 nmol of each primer. The cycling parameters were 94 °C for 3 min for denaturation; followed by 35 cycles at 94 °C for 1 min, 55 °C for 1 min, 72 °C for 2 min; and a final extension at 72 °C for 10 min. The amplified products were separated by 8% PAGE gel as described by Hu *et al.* [28].

### 2.4. Agronomic Performance Observations

Field agronomic trait observations were performed at the Xindu Experimental Station, Chengdu, China during the 2012–2015 wheat-growing season. A post-harvest re-growth (PHR) habit displays a second phase of tiller initiation after the sexual cycle of the first phase is completed [29]. After harvesting, the 40 cm stubble of the lines was left in the field for evaluation of re-growth. Either a crown or tiller emerging from the soil surface was taken to be regrowth, with regrowth expressed as a percentage of PHR measured one month after harvest.

## 3. Results

### 3.1. FISH Karyotype of D. breviaristatum Chromosomes in TDH-2

**Figure 1:**
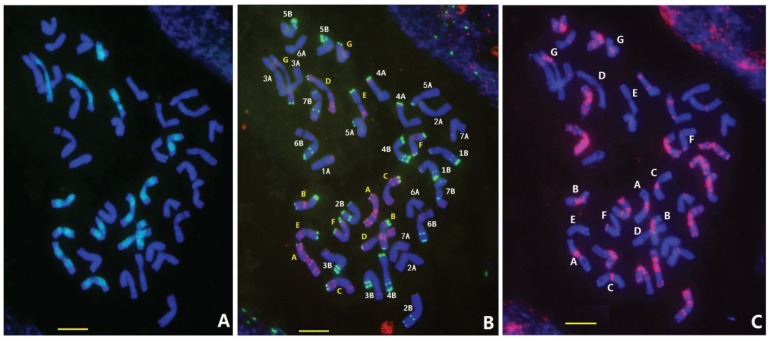
Sequential fluorescence *in situ* hybridization FISH of wheat—*D. breviaristatum* partial amphiploid (TDH-2) with probes pDb12H (**A**), Oligo-pSc119.2 (**green**) and Oligo-pTa535 (**red**) (**B**) and Oligo-(GAA)_7_ (**C**) (**red**). The bars indicated 10 µm.

**Figure 2:**
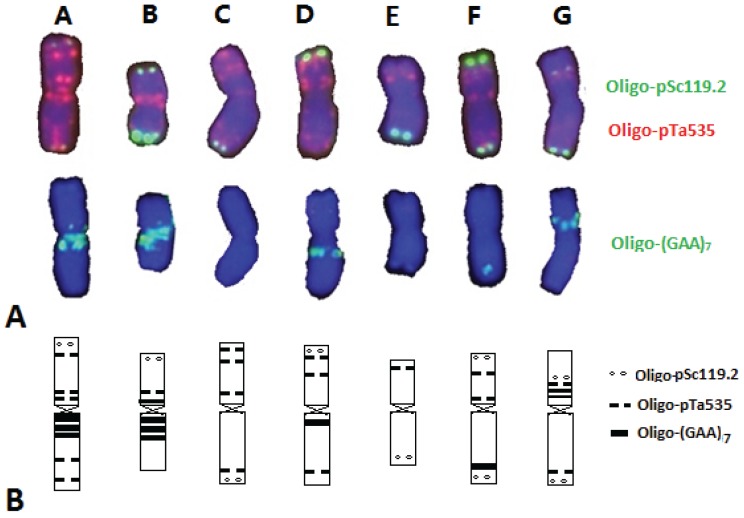
Karyotype (**A**) and ideogram (**B**) of *D. breviaristatum* chromosomes presents in the wheat—*D. breviaristatum* partial amphiploid (TDH-2).

In order to establish the FISH karyotype of *D. breviaristatum* chromosomes, a partial amphiploid between wheat—*D. breviaristatum* [9], the mitotic metaphase chromosomes of TDH-2 that were hybridized using the Oligo-pSc119.2, Oligo-pTa535, Oligo-(GAA)_7_ and pDb12H probes through sequential multicolor-FISH (Figure 1). As shown in Figure 1A, strong hybridization signals of the *Dasypyrum* specific probe pDb12H [23] were observed on all 14 chromosomes of TDH-2, indicating that they are *D. breviaristatum* chromosomes. Subsequently, Oligo-pSc119.2 and Oligo-pTa535 probes were also used to identify the *D. breviaristatum* chromosomes (temporarily designed from A to G) of the same metaphase of the TDH-2 partial amphiploid (Figure 1B). We found that the signals using Oligo-pSc119.2 probe were mainly located on the terminal sites of one arm in four pairs of chromosomes (C, D, F and G), and both arms in two pairs of chromosomes (B and F) (Figure 1B and Figure 2A). The hybridization signals of Oligo-pTa535 were distributed on all the chromosome arms of *D. breviaristatum*, including signals at the terminal, sub-terminal or interstitial sites and occasionally at centromeric positions (Figure 1B and Figure 2A). The Oligo- (GAA)_7_ probe hybridized to five pairs of *D. breviaristatum* chromosomes (A-B, D, F-G) at their centromeric regions or sub-terminal regions, while two pairs of chromosomes (C and E) were free of Oligo- (GAA)_7_ hybridization sites (Figure 1C and Figure 2A). Based on the distribution of the above four probes, the FISH karyotype of the seven pairs of *D. breviaristatum* chromosomes in TDH-2 was obtained (Figure 2B). Compared with the reported FISH karyotype of wheat and other Triticeae genomes [16,17,24,25], we conclude that FISH can precisely identify the *D. breviaristatum* chromosomes in a wheat background.

### 3.2. FISH of D2150 and D2146

**Figure 3:**
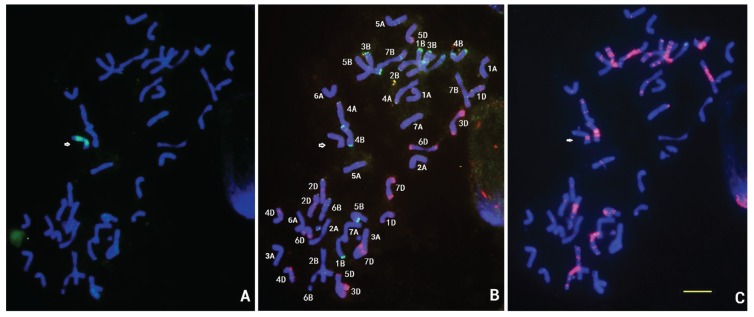

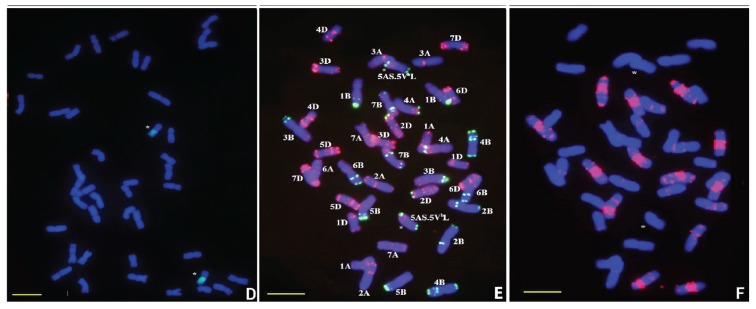
FISH of wheat—*D. breviaristatum* derivative lines. (**A**–**C**) wheat-monosomic addition line for chromosome 5V^b^ of *D. breviaristatum* (D2150) and (**D**–**F**) 5AS.5V^b^L translocation line (D2146). The probes pDb12H (**A**,**D**) and Oligo-pSc119.2 (**B**,**E**) are showed in green. The probes Oligo-pTa535 (**B**,**E**) and Oligo- (GAA)_7_ (**C**,**F**) are showed in red. Arrows and stars show the *D. breviaristatum* chromatin, and bars indicate 10 µm.

The probes Oligo-pSc119.2, Oligo-pTa535, Oligo-(GAA)_7_ and pDb12H were used to identify the chromosomes in metaphase spreads of wheat—*D. breviaristatum* D2146 and D2150 lines (Figure 3A). FISH with the pDb12H probe revealed that D2150 had 43 chromosomes including a *D. breviaristatum* chromosome (Figure 3A). The sequential FISH using Oligo-Sc119.2 and Oligo-pTa535 (Figure 3B), as well as the Oligo-(GAA)_6_ (Figure 3C), suggested that in the D2150 line, the added *D. breviaristatum* chromosome was identical to the chromosome G of TDH-2 (Figure 2). FISH by pDb12H indicated that D2146 carried a pair of wheat—*D. breviaristatum* translocated chromosomes (Figure 3D). The FISH with probes Oligo-pSc119.2 and Oligo-pTa535 (Figure 3E), and Oligo-(GAA)_6_ (Figure 3F), indicated that the translocated chromosome in D2146 line showed one weak Oligo-pSc119.2 band at the end of the short arm while the long arm of *D. breviaristatum* showed both a strong pSc119.2 and faint Oligo-pTa535 sites at the terminal regions. We deduced that the short arm showed the typical FISH pattern of 5AS and the long arm resembled that of chromosome G of *D. breviaristatum*.

### 3.3. Molecular Marker Analysis

**Figure 4:**
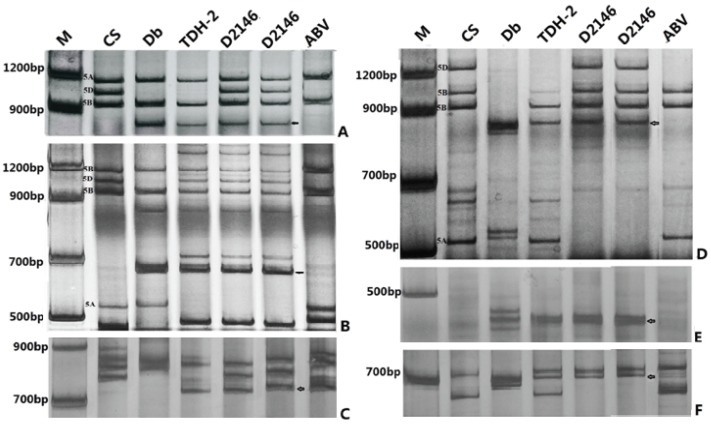
PCR amplification of molecular markers in wheat—*D. breviaristatum* lines. (**A**) TNAC1485; (**B**) TNAC1554; (**C**) BE443610; (**D**) TNA1567; (**E**) BE445380 and (**F**) BE604833. CS: “Chinese Spring” wheat; Db: *D. breviaristatum*; TDH-2: wheat—*D. breviaristatum* partial amphiploid; D2146: 5AS.5V^b^L translocation line; ABV: *Triticum turgidum*, *D. villosum* amphiploid. The arrows indicate the *D. breviaristatum* specific bands.

**Figure 5:**
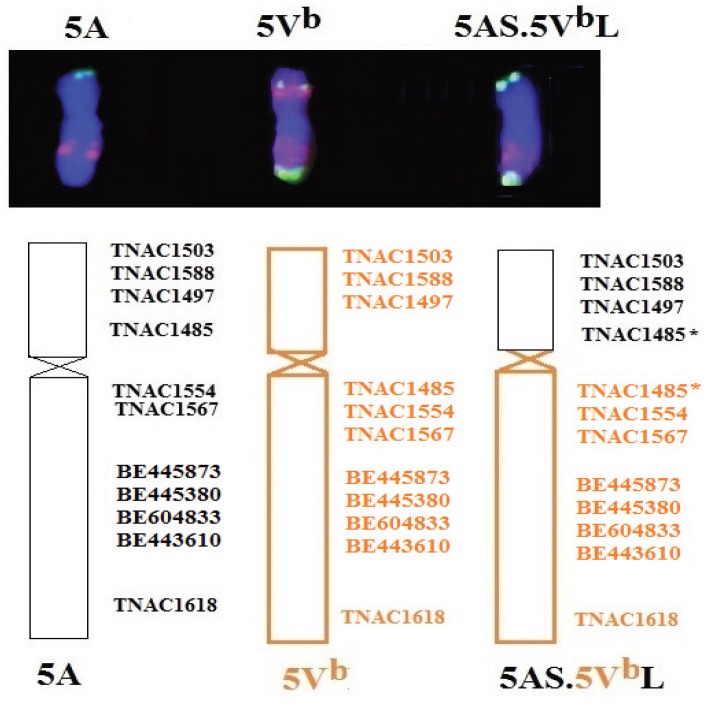
FISH karyotypes and molecular markers distributed on chromosomes 5A, 5V^b^, and 5AS.5V^b^L translocation. The stars indicates the duplicated markers on chromosome 5AS.5V^b^L.

In order to determine the linkage group of the *D. breviaristatum* chromatin in D2146, molecular markers based on the syntenic regions between wheat EST and rice genomic DNA sequences were used to identifying alien fragments corresponding to the wheat linkage group(s) [30,31,32]. A total of 12 PLUG markers from wheat homologous group 5 [26] and 22 *Hordeum californicum* chromosome 5H^c^ specific markers [27] were used. The markers were tested on D2146 and its parents (MY11 and TDH-2) as well as *D. breviaristatum*. We found that 13 markers generated specific bands from *D. breviaristatum* and TDH-2. Three markers were assigned to the short arm and 10 markers were located on long arm of *D. breviaristatum* chromosome 5V^b^ (Table 1). As shown in Figure 4, the PLUG markers TNAC1554 and TNAC1567 amplified fragments from the long arm of chromosomes 5A, 5B, and 5D of common wheat CS. The chromosome 5AL specific fragments were absent in D2146, while the *D. breviaristatum* specific bands appeared in D2146. These results suggested that the 5AS chromosome arm was translocated to the 5V^b^L. However, marker TNAC1485 simultaneously amplified 5AS and 5V^b^L specific bands in the D2146 translocation line (Figure 4A). As shown in Figure 5, we conclude that D2146 translocation line may contain a putative duplicated fragment of homologous group 5 from wheat and *Dasypyrum* chromosomes 5V^b^.

**Table 1:** The PCR primers used in this study.

Markers	Homoeologous Relationship	Primer Sequences	Enzymes	*Dasypyrum* Specific Bands
TNAC 1485 ^a^	5AS,5BS, 5DS	F: CCCAAGTTCACTAACTTCGTTG	*Taq* I	5V^b^L
		R: AAATAGTCCTGCATATCTCCTGT		
TNAC 1497 ^a^	5AS,5BS, 5DS	F: ATCAAACCTGACGGTGTTCAG	*Taq* I	5V^b^S
		R: CATGCAGACTACAGGTCCAGA		
TNAC1503 ^a^	5AS,5BS, 5DS	F: TGAGGTTGGTTCTCATCTGGA	*Taq* I	5V^b^S
		R: CGTTGGAAACAATCTGAATGG		
TNAC1588 ^a^	5AS,5BS, 5DS	F: AAATCAGCAGGTGGCCAGTAT	*Taq* I	5V^b^S
		R: AAATGGCGCACCATACTCAAG		
TNAC1540 ^a^	5AL,5BL, 5DL	F: AACCTCAAGCACTGTCAGCAT	*Hea* III	5V^b^L
		R: TTGCAGATCCTCTCAATCTCG		
TNAC 1554 ^a^	5AL,5BL, 5DL	F: TTGCTAGCTCAGCACAGTTTG	*Taq* I	5V^b^L
		R: TTCTTGGTCACTCTGAGCGTA		
TNAC1559 ^a^	5AL,5BL, 5DL	F: AAACAAGGCCCTGAAACACTT	*Hea* III	5V^b^L
		R: CATTGTCAGGCTATGGGACAT		
TNAC 1567 ^a^	5AL,5BL, 5DL	F: ATGTTGGCTTTATACCAATGC	*Taq* I	5V^b^L
		R: AGGTGCGGCTTCACTATCTTT		
TNAC 1618 ^a^	5AL,5BL, 5DL	F: GTTGGCTGTTGATGGTAAGGA	*Taq* I	5V^b^L
		R: GGAGGCCACCAACTAATGTTT		
BE445873 ^b^	5AL,5BL,5DL	F: ATCTCGACAAAGATCAAGCA	*-*	5V^b^L
		R: CGAGAAGTTCCATCTCATTG		
BE445380 ^b^	5AL,5BL	F: GCTACCACAGTTGCTACAGG	*-*	5V^b^L
		R: ATCGACGTAACACGAATCAC		
BE604833 ^b^	5AL	F: GCAGATTCACCCACTCTGTA	*-*	5V^b^L
		R: ATACGCGGTCACATCATAAA		
BE443610 ^b^	5AL,5BL,5DL	F: ACCAATGAAGGACCATCTCT	*-*	5V^b^L
		R; CATTTCTCAGCTTGTCCAAC		

(Note: ^a^ Ishikawa *et al.* [26]; ^b^ Fang *et al.* [27]).

### 3.4. Agronomic Traits Observation

A set of agronomic traits were measured on 15 plants of the *D. breviaristatum* 5V^b^ monosomic addition line, 5AS.5V^b^L translocation line, wheat MY11 and the wheat—*D. breviaristatum* partial amphiploid (Table 2). Relative to the MY11 recurrent parent, all 5V^b^ lines had reduced plant height, suggesting that chromosome 5V^b^ carries a dwarfing gene(s) expressed in the wheat background. No significant differences were found for the length of spikes in the 5V^b^ monosomic addition line, 5AS. 5V^b^L translocation line and the wheat control. The 5AS. 5V^b^L translocation line had a decreased number of spikelets per spike and a 1000-kernel weight compared to its wheat parent indicating that the translocation may have an unfavorable effect on grain yield relative to wheat lines.

The wheat—*D. breviaristatum* partial amphiploid (TDH-2), *D. breviaristatum* 5V^b^ monosomic addition (D2150), and 5AS. 5V^b^L translocation line (D2146) were found to have PHR habits under field conditions, while the parent MY11 has no PHR habit (Table 2). This result indicates that *D. breviaristatum* 5V^b^L may contain a gene(s) responsible for the PHR habit in annual wheat background.

**Table 2:** Agronomical traits of wheat—*D. breviaristatum* 5V ^b^ derivatives.

Genotype	Plant Height (cm)	Length of Spike (cm)	No. of Spikelet	No. of Spikes	1000-Kernel Weight (g)	Re-Growth Score
MY11	86.5 ± 1.2a	10.5 ± 0.5b	20.6 ± 0.2a	4.2 ± 0.2b	40.4 ± 1.0a	0
TDH-2	70.0 ± 4.8b	14.2 ± 0.5a	16 .8 ± 0.3b	7.5 ± 0.5a	16.5 ± 0.6c	86
D2146	65.0 ± 3.0b	10.0 ± 0.4b	15 .0 ± 1.5b	3.0 ± 0.5b	31.7 ± 1.7b	56
D2150	77.3 ± 3.5ab	11.0 ± 0.5b	19.1 ± 1.5a	3.9 ± 0.5b	39.7 ± 0.8a	78

(Note: Values with the same letter in the same column do not differ significantly at *p* < 0.05).

## 4. Discussion

Fluorescence *in situ* hybridization (FISH) and genomic *in situ* hybridization (GISH) have been most useful techniques for investigating wheat—alien derivatives [13,33]. However, the conventional FISH protocols based on probe labeling, hybridization and detection were somewhat time-consuming and expensive [34,35]. The simpler and more efficient technique based on synthetic labeled oligonucleotides combined with non-denaturing FISH (ND-FISH) analysis were recently developed [24,36,37,38]. The synthetic oligonucleotides have been successfully used in FISH experiments, including the SSRs oligonucleotides and conserved nucleotides representing repetitive sequences, for karyotyping wheat, barley and rye chromosomes [39,40,41]. Recently, chromosome-specific painting in plant species using synthetic bulked oligonucleotides was also established [42]. In the present study, we detected the wheat and *Dasypyrum* chromatin using synthetic labeled oligonucleotides by ND-FISH with chromosome preparation of both a conventional squash method (Figure 1 and Figure 3A–C) and a nitrous oxide treated drop method (Figure 3D–F). In combination with *Dasypyrum* specific LTR probe pDb12H [23], we used the synthetic oligonucleotide probes, Oligo-pTa535, Oligo-pSc119.2, and Oligo-(GAA)_7_ [23], to develop a high resolution FISH karyotype of *D. breviaristatum* chromosomes in TDH-2. The FISH karyotypes can be used to the precisely locate the *Dasypyrum* chromatin in a wheat background. Comparing the FISH patterns of *D. breviaristatum* chromosomes present in the wheat—*D. breviaristatum* partial amphiploid (TDH-2) we found that the added *D. breviaristatum* chromosome in the D2150 line was identical to chromosome G of TDH-2 and that the fragment of *D. breviaristatum* present in the D2146 translocation line resembled the long arm of the chromosome G. Grosso *et al.* [17] used the simple sequence repeat (GAA)*_n_* as a FISH probe, to characterize the individual *D. villosum* chromosomes except for chromosome 1V. Recently, Zhang *et al.* [16] investigated the FISH distribution patterns of three repeated DNA sequences, pSc119.2, pAs1, 45S rDNA and 5S rDNA in the individual *D. villosum* chromosomes of *D. villosum* wheat addition and translocation lines. Compared with the FISH pattern of *D. villosum* chromosomes by Zhang *et al.* [16], we found that the seven pairs of *D. breviaristatum* chromosomes in the wheat—*D. breviaristatum* partial amphiploid displayed unique FISH patterns*.* Two pairs of *D. breviaristatum* chromosomes were lacking of (GAA)*_n_* signals (Figure 2), the other five *D. breviaristatum* chromosomes pairs showed weaker (GAA)*_n_* signals than those of *D. villosum* chromosomes [17]. Moreover, the terminal regions of the short arms of three *D. breviaristatum* chromosomes showed strong pSc119.2 signals, while almost all *D. villosum* chromosome short arms have the pSc119.2 signals [16]. The results suggested that the accumulation of the repetitive sequences in *D. breviaristatum* chromosomes was less than in *D. villosum* chromosomes. The results were consistent with the evolutionary studies on the *D. breviaristatum* and *D. villosum* chromosomes by cytogenetic and molecular evidence [43,44], and supported the idea that *D. breviaristatum* was ancestral to the *D. villosum* species [3,5]*.*

The production of compensating Robertsonian translocations is an important step for the evaluation of the breeding value of alien genetic materials [8]. Liu *et al.* [34] reported a set of wheat—*D. villosum* compensating Robertsonian translocations including a line TA5638 with T5DL·5V#3S translocation between 5V and 5D. Zhang *et al.* [45] irradiated whole-arm wheat—*D. villosum* T5VS·5DL translocation line, and produced six homozygous small segment translocation lines with different fragment sizes of 5VS, and a 5VS-6AS·6AL terminal translocation. In the present study, we produced a line (D2150) with a *D. breviaristatum* 5V^b^ chromosome in monosomy and a homozygous T5AS·5V^b^L translocation line (D2146). Based on the molecular markers analysis, nine of 10 markers validated the introgression of 5V^b^L. However, the specific amplification of marker TNAC1485 appeared in both chromosome 5AS and 5V^b^L arms (Figure 4A). It is suggested that the homologous duplication of the segments has occurred in this region in the T5AS·5V^b^L translocation lines. Recently, Li *et al.* [32] reported that the rye (*Secale cereale* L.) chromosome 5RL also contained homologous 5S wheat segments. These authors found that TNAC1485 marker was also located on rye chromosome 5RL. It is likely that the rearrangement occurred between the ancestral group 5 in *Secale* and in *Dasypyrum* chromosomes. Further evidence is needed to clarify the detail changes, possibly by inversion or centromeric movement during evolution by comparative genomic studies between wheat and related species.

Based on the agronomic traits evaluated (Table 2) it is likely that incomplete compensation of chromosome 5AS.5V^b^L in the D2146 line may cause inferior agronomic traits, such as reduced grain weight and spikelet number, compared with the wheat parent. Amphiploids and addition lines among wheat and some perennial species have shown the post-harvest re-growth habit (PHR) which has been investigated to produce potentially perennial wheat [46]. So far, wheat—*Thinopyrum* partial amphiploids [20,21] and wheat—*Th. elongatum* chromosome 4E addition lines [29] have been reported to express the PHR traits from these alien species in a wheat background [47]. As a perennial species, *D*. *breviaristatum* showed a strong perennial growth habit. The wheat—*D. breviaristatum* partial amphiploid (TDH-2) and the 5V^b^ derived lines (D2146 and D2150) also showed the PHR trait which putatively originated from *D. breviaristatum* parent. These results suggest that the PHR trait could be controlled by genes located on *D. breviaristatum* 5V^b^L chromosome arm. The lines with PHR habits will be useful for the development of perennial grain crops for feeding the animals.

## 5. Conclusions

*Dasypyrum breviaristatum* was a perennial species with a post-harvest re-growth character. Based on the molecular and cytogenetic studies, novel wheat—*D. breviaristatum* 5V^b^ chromosome addition and 5AS.5V^b^L translocation line were characterized. New *D. breviaristatum* 5V^b^ specific molecular markers were also produced. The wheat—*D. breviaristatum* derivatives and molecular markers may be favorable for future use of *D. breviaristatum* resources for development of perennial growth wheat lines.
